# Gastro-oesophageal reflux disease in type 2 diabetics: symptom load and pathophysiologic aspects - a retro-pro study

**DOI:** 10.1186/1471-230X-13-132

**Published:** 2013-08-23

**Authors:** Regina Promberger, Johannes Lenglinger, Otto Riedl, Gernot Seebacher, Wolf Hans Eilenberg, Johannes Ott, Franz Martin Riegler, Michael Gadenstätter, Christoph Neumayer

**Affiliations:** 1 Division of General Surgery, Department of Surgery, Medical University of Vienna, Vienna, Austria; 2 Karl Landsteiner Institute for Clinical Surgery, General Hospital of Krems, Krems, Austria; 3 Division of Plastic and Reconstructive Surgery, Department of Surgery, Medical University of Vienna, Vienna, Austria; 4 Department of Gynecology and Obstetrics, Medical University of Vienna, Vienna, Austria; 5 Division of Vascular Surgery, Department of Surgery, Medical University of Vienna, Vienna, Austria

**Keywords:** Diabetes, GERD, Dysphagia, Heartburn, Hiatal hernia

## Abstract

**Background:**

Information about gastro-oesophageal reflux disease (GERD) in patients with Diabetes mellitus type 2 (T2D) is scarce, although the incidence of both disorders is increasing.

We aimed to determine GERD symptoms and their underlying pathophysiologic characteristics in T2D patients.

**Methods:**

This “retro-pro” study compared 65 T2D patients to a control group of 130 age- and sex-matched non-diabetics. GERD was confirmed by gastroscopy, manometry, pH-metry and barium swallow.

**Results:**

In patients with T2D compared to controls, dysphagia (32.3% vs. 13.1%; p = 0.001) and globus sensation (27.7% vs. 13.8%; p = 0.021) were found more frequently, whereas heartburn (76.9% vs. 88.5%; p = 0.046) and regurgitation (47.7% vs. 72.3%; p = 0.001) were predominant in non-diabetics. Despite higher body mass indices (31.1 ± 5.2 vs. 27.7 ± 3.7 kg/m^2^; p < 0.001), hiatal hernia was less frequent in T2D patients compared to controls (60.0% vs. 90.8%, p < 0.001). Lower oesophageal sphincter (LES) pressure was higher in patients with T2D (median 10.0 vs. 7.2 mmHg, p = 0.016). DeMeester scores did not differ between the groups. Helicobacter pylori infections were more common in T2D patients (26.2% vs. 7.7%, p = 0.001). Barrett metaplasia (21.5% vs. 17.7%), as well as low- (10.8% vs. 3.8%) and high-grade dysplasia (1.5% vs. 0%) were predominant in T2D patients.

**Conclusions:**

T2D patients exhibit different GERD symptoms, higher LES pressures and a decreased prevalence of hiatal hernia than non-diabetics, which may be related to worse oesophageal motility and, thus, a more functional rather than anatomical cause of GERD. Low-grade dysplasia was more than twice as high in T2D than in non-diabetics patients.

**Trial registration:**

Ethics committee of the Medical University of Vienna, IRB number 720/2011.

## Background

Gastro-oesophageal reflux disease (GERD) is one of the most common disorders of the upper gastrointestinal tract in developed countries. Up to 40% of the adult population suffers from reflux symptoms [1]. At the same time, diabetes mellitus (DM), especially DM type 2 (T2D), which accounts for up to 95% of diabetes cases, is dramatically increasing worldwide. In 2010, 284.8 million people were affected. It has been estimated that that number will rise to 438.7 million diabetic adults in the year 2030 [2]. Thus, both disorders are of increasing medical and socioeconomic interest.

T2D has been described as a possible risk factor for the development of GERD [3-6]. More recently, it has been suggested that the metabolic syndrome defined by visceral fat accumulation, dyslipidaemia, hypertension, and hyperglycaemia correlates with the occurrence of GERD [7]. As recently reviewed, several pathophysiologic factors may explain this finding: (i) hyperglycaemia causes increased gastric H^+^ secretion, higher levels of bile acids, and reduced bicarbonate levels; (ii) delayed oesophageal and gastric emptying, increased rates of transient lower oesophageal sphincter (LES) relaxations, and decreased LES pressure has been reported during hyperglycaemia in patients with T2D; and, (iii) hiatal hernia and increased obesity rates may also contribute to the development of GERD in these patients [8].

However, many of the reviewed studies did not discriminate between GERD-specific symptoms and general upper gastrointestinal symptoms, which are frequently found in diabetics [8]. Moreover, most studies were based on GERD questionnaires and did not determine GERD using standard pH-metry and manometry [3-5,9,10]. To date, no study exists about GERD and GERD-specific symptoms in patients with T2D that has included upper gastrointestinal endoscopy, barium oesophagogram, manometry, and 24-hour oesophageal pH-monitoring concurrently.

The aim of this study was to investigate GERD-specific symptoms and reflux parameters in patients with T2D using these standard diagnostic tools. Second, the results were compared to non-diabetic GERD-patients in order to explore possible diabetes-related differences. Special emphasis was placed on extra-oesophageal GERD symptoms that have not been described for diabetics thus far.

## Methods

### Patient selection and assessment of symptom load

All patients referred to the motility laboratories of our institutions for symptoms suggestive of GERD between January 2007 and January 2009 were considered eligible for this “retro-pro” case control study [11]. Exclusion criteria were a body mass index (BMI) >35 kg/m^2^, type 1 (juvenile) diabetes, oesophageal motility disorders other than ineffective oesophageal motility, and a history of oesophageal or gastric surgery. The study group consisted of patients with T2D and GERD-related symptoms. The diagnosis of T2D was based on the criteria established by the American Diabetes Association [12]. Of a total of 588 patients, a control group of 130 non-diabetic GERD patients (22.1%) was selected using age and sex as matching criteria. A detailed history was taken from all patients and they were asked to answer a standardized questionnaire about upper gastrointestinal symptoms before oesophageal function tests. Patients were questioned regarding the presence of upper gastrointestinal symptoms, namely heartburn, epigastric pain, regurgitation, respiratory symptoms, odynophagia, globus sensation, dysphagia, flatulence, chronic cough, hoarseness, bronchitis, and bronchial asthma. Bronchitis and bronchial asthma were only accepted as co-morbidities if a specialist in respiratory medicine had diagnosed them. All patients’ medication was also assessed, with a focus on proton-pump inhibitors, pro-kinetics, and anti-depressants.

Data were prospectively gathered by a research assistant and entered in a database. The study was approved by the ethics committee of the Medical University of Vienna (IRB number: 720/2011). The study was carried out in accordance with the “Good Scientific Practice” Standards of the Medical University of Vienna, which are based on the ethical standards of the Helsinki Declaration of 1975. Written informed consent was obtained from all study participants.

### Oesophageal testing

All patients underwent upper gastrointestinal endoscopy, stationary oesophageal manometry, barium oesophagogram, and 24-hour oesophageal pH-monitoring. Thereby, GERD was confirmed for all patients of both the T2D and the control group. Upper gastrointestinal endoscopy was routinely performed with sedation on a Fujinon Video Endoscope System (Fujifilm Holdings Corporation, Tokyo, Japan). Esophagitis was graded using the Savary and Miller classification system [13]. A hiatal hernia was diagnosed when the difference between the position of the crural impression and the commencement of gastric-type folds was 2 cm or more. The size of the hiatal hernia was noted. Standardized biopsies were obtained from the oesophagus, the squamocolumnar junction, and the gastric antrum. Visible tongues or circular segments of columnar epithelium were biopsied. A diagnosis of intestinal metaplasia (Barrett’s oesophagus) was made if the presence of well-defined goblet cells within columnar epithelium was confirmed by a pathologist who specialized in gastrointestinal disorders [14]. Laryngeal lesions, including inflammation, edema, leukoplakia, and ulcerations were also diagnosed or ruled out, either in the course of upper gastrointestinal endoscopy and/or laryngoscopy. An oto-laryngologist saw these patients and confirmed the lesions.

All patients underwent standard, stationary, pull-through oesophageal manometry using the Sandhill EFT Impedance Manometry System ® (Sandhill Scientific, Colorado, USA) with Bioview Software ® (Sandhill Scientific, Colorado, USA). LES resting pressure and swallow-induced relaxation was measured immediately distal to the respiratory inversion point. LES was considered mechanically defective if the mean end-expiratory pressure was below 8 mm Hg and/or a total LES length < 2 cm or an abdominal length < 1 cm was encountered [15,16]. Oesophageal body motility was investigated with ten water swallows of 5 ml at 30-second intervals. Propagation and amplitudes of swallow-induced contractions were recorded in the proximal, middle, and distal third of the oesophageal body. A contraction wave was considered to be ineffective if one or more of the following criteria applied: 1) amplitude in the distal oesophagus below 30 mmHg; 2) simultaneous propagation (> 20 mm/sec); or 3) interrupted or dropped contraction waves (contraction amplitudes < 15 mmHg). Impaired oesophageal motility was diagnosed if more than 20% of oesophageal contraction waves fulfilled these criteria [17].

Twenty-four-hour oesophageal pH-monitoring was performed using the Sandhill GER Impedance pH Metry ® device (Sandhill Scientific, Colorado, USA) as described previously [18]. In short, a single-use catheter fitted with an antimony electrode was passed transnasally and positioned 5 cm proximal to the upper border of the LES. A calibration of buffer solutions of pH 1 and pH 7 was performed prior to each study. Endoluminal pH was recorded for 20–24 hours at a frequency of 0.25 Hz, and data were stored in a portable data logger. Patients were instructed to follow their routine activities and to consume their usual diet. The patients recorded meal periods and times spent in recumbent positions. Sampled data were transferred to a computer for analysis using DeMeester’s composite score as the main variable. A score of more than 14.7 indicated increased acid reflux. All medications that could possibly interfere with oesophageal motor function (i.e., metoclopramide, cisapride, nitrates, beta-agonists, and calcium-channel blocking agents) or gastric acid secretion were discontinued at least seven days before the studies. The use of sucralfate was permitted until one day before reflux monitoring.

In addition, barium swallows, i.e., double-contrast oesophagographies, were performed in all patients through biphasic examination with upright air-contrast and prone single-contrast views of the oesophagus. The occurrence of spontaneous gastro-oesophageal reflux was assessed, and the presence and size of hiatal hernias were recorded.

### Statistical analysis

Values are expressed as mean ± standard deviation (SD), or median and interquartile range [IQR], if appropriate. Concerning general patient characteristics, the chi-square test and, if appropriate, Fisher’s exact test, were used to compare proportions between groups. To compare differences, t-tests and u-tests were used. To compare proportions and differences in outcome parameters between the groups, univariate logistic regression models were used. For these, p-values of the likelihood ratio tests, odds ratios (OR) and 95% confidence intervals (95% CI) are provided. Correlations were tested using Spearman rank correlations. P values <0.05 were considered statistically significant. The software SPSS 17.0 (SPSS Inc, Chicago, 1993–2007) was used for all statistical analyses.

## Results

### Patient characteristics

One-hundred-thirty non-diabetic GERD-patients were enrolled in the study as a control-group. General patient characteristics are summarized in Table 1. BMI levels were significantly higher in diabetic patients. When focusing on laboratory parameters, total cholesterol levels were higher in the control group, whereas HbA1c and triglyceride levels were higher in patients with T2D.

**Table 1:** Comparison of general patient characteristics and laboratory parameters between GERD patients with and without T2D

	**T2D GERD patients**	**Non-diabetic GERD patients**	**p**
Patients (n)	65	130	---
Females (n [%])	29 [44.6%]	58 [44.6%]	n.s.*
Age (years, mean ± SD)	58.6 ± 9.2	58.5 ± 8.8	n.s.**
BMI (kg/m^2^, mean ± SD)	31.1 ± 5.2	27.7 ± 3.7	<0.001**
Smoking (n [%])	18 [27.7%]	12 [10.1%]	0.002*
Duration of GERD symptoms (years, median [IQR])	5.0 [3.0;10.0]	5.0 [2.0;11.0]	n.s.***
HbA1c (%)	7.2 ± 1.1	5.3 ± 0.9	<0.001**
Total cholesterol (mg/d, mean ± SD)	204.2 ± 44.9	217.3 ± 38.0	0.013***
LDL* (mg/dl, mean ± SD)	126.6 ± 32.6	134.8 ± 36.9	n.s.***
HDL^#^ (mg/dl, mean ± SD)	56.3 ± 16.0	60.2 ± 18.3	n.s.***
Triglycerides (mg/dl, mean ± SD)	183.0 ± 118.9	128.8 ± 66.4	<0.001***
Blood urea nitrogen (mg/dl, mean ± SD)	15.1 ± 4.9	13.7 ± 4.5	n.s.***
Creatinine (mg/dl, mean ± SD)	1.0 ± 0.2	0.9 ± 0.1	n.s.***

*n* number, *SD* Standard deviation, *IQR* Interquartile range, *BMI* Body mass index, *n.s.* Not significant; * chi-square test, ** t-test, *** u-test, p p-value.

No differences were found between diabetics and non-diabetics concerning the use of proton pump inhibitors and pro-kinetics (59/65 [90.8%] vs. 118/130 [90.8%], p > 0.05; and 4/65 [6.2%] vs. 10/130 [7.7%], p > 0.05, respectively). Anti-depressants were used more frequently in diabetics than in non-diabetics (14/65 [21.5%] vs. 7/130 [5.4%], p = 0.001, respectively).

When focusing on the characteristics of patients with T2D only, 56 subjects had T2D for a median duration of 5.0 years (IQR 3.0-10.0 years; 29 women, 36 men). Neither the duration of T2D, nor the duration of GERD correlated with HbA1c levels (r = 0.254, p = 0.061 and r = −0.063, p = 0.617; respectively).

All diabetic subjects were treated dietetically. In addition, 49 patients (75.4%) used hypoglycaemic agents, such as metformin (40/65, 61.5%), gliclacid (6/65, 9.2%), glibenclamid (2/65, 3.1%), and acarbose (1/65, 1.5%). Twelve patients (18.5%) were insulin-dependent. The co-morbidities of T2D patients comprised arterial hypertension in 58.5% (38/65), peripheral neuropathy in 32.3% (21/65), diabetic retinopathy in 13.8% (9/65), peripheral arterial disease in 12.3% (8/65), and diabetic nephropathy in 6.2% (4/65).

### GERD-related symptoms

Table 2 provides data about GERD-related symptoms. In short, patients with T2D reported dysphagia and globus sensation more frequently, whereas heartburn, regurgitation, and flatulence were predominant in non-diabetics. The prevalence of respiratory symptoms did not differ between both groups. When comparing the sum of GERD symptoms between patients with and without T2D, there was no essential difference (median 3, IQR 2–5 vs. median 4, IQR 3–5, respectively; p = 0.210). Likewise, diabetic patients with neuropathia and those without did not differ concerning the sum of GERD symptoms (median 3, IQR 2–4, vs. median 3, IQR 2–5, respectively; p = 0.411). The duration of diabetes had no impact on GERD symptoms either.

**Table 2:** Upper gastrointestinal symptoms in diabetic and non-diabetic GERD patients

	**T2D GERD patients**	**Non-diabetic GERD patients**	**OR (95% CI)**	**p**
	**n = 65**	**n = 130**		
Heartburn	50 (76.9)	115 (88.5)	0.47 (0.21;0.99)	0.046
Epigastric pain	40 (61.5)	85 (65.4)	0.85 (0.46;1.57)	n.s.
Regurgitation	31 (47.7)	94 (72.3)	0.36 (0.20;0.67)	0.001
Respiratory symptoms	40 (61.5)	82 (63.1)	0.94 (0.51;1.73)	n.s.
Odynophagia	6 (9.2)	9 (6.9)	1.37 (0.47;4.02)	n.s.
Globus sensation	18 (27.7)	18 (13.8)	2.38 (1.14;4.80)	0.021
Dysphagia	21 (32.3)	17 (13.1)	3.40 (0.90;12.79)	0.001
Flatulence	13 (20.0)	59 (45.4)	0.30 (0.15;0.61)	0.001
Chronic cough	28 (43.1)	59 (45.4)	0.91 (0.50;1.66)	n.s.
Hoarseness	17 (26.2)	32 (24.6)	1.08 (0.55;20.14)	n.s.
Bronchitis	10 (15.4)	22 (16.9)	0.89 (0.39;2.02)	n.s.
Asthma bronchiale	9 (13.8)	28 (21.5)	0.59 (0.26;1.33)	n.s.

*n.s.* not significant, p p-value.

Data are provided as n(%), multiple citations possible; all parameters were tested using univariate logistic regression models.

### Laryngeal findings and oesophageal testing

Laryngeal lesions, including inflammation, oedema, leukoplakia, and ulcerations were observed more frequently in patients with T2D (16.9% vs. 7.0% in non-diabetics, p = 0.032).

Results of oesophageal testing are given in Table 3. When focusing on findings with differences between diabetic and non-diabetic patients only, upper GI endoscopy revealed a hiatal hernia in 60.0% of patients with T2D compared to 90.8% of non-diabetics (p < 0.001). The sizes of hiatal hernias did not differ significantly between the groups (median 4 cm [IQR 3–5] for patients with T2D vs. median 5 cm [IQR 4–7] for non-diabetics, p = 0.060). An analysis of barium esophagograms revealed hiatal hernias more frequently in non-diabetics. Helicobacter pylori infections were more common in patients with T2D. Manometry revealed a significantly higher median pressure of LES for diabetics.

**Table 3:** Results of oesophageal testing

	**T2D GERD patients**	**Non-diabetic GERD patients**	**OR (95% CI)**	**p**
	**n = 65**	**n = 130**		
*Upper GI endoscopy*				
Hiatal hernia (n [%])	39 [60.0]	118 [90.8]	0.49 (0.23;0.98)	<0.001
Oesophagitis (n [%])	30 [46.2]	43 [33.1]	1.86 (1.01;3.43)	n.s.
Barrett metaplasia (n [%])	11 [16.9]	35 [26.9]	0.58 (0.27;123)	n.s.
*Histology*				
Esophagitis (n [%])	38 [58.5]	73 [56.2]	1.32 (0.73;2.42)	n.s.
Barrett metaplasia (n [%])	14 [21.5]	23 [17.7]	1.42 (0.67;3.02)	n.s.
Low-grade dysplasia (n [%])	7 [10.8]	5 [3.8]	3.80 (1.07;13.51)	n.s.
High-grade dysplasia (n [%])	1 [1.5]	0 [0]	3.28*e^9^ (0;inf)	n.s.
Helicobacter pylori (n [%])	17 [26.2]	10 [7.7]	4.72 (1.97;11.32)	0.001
*Manometry*				
LES pressure (mm Hg, median [IQR])	10.0 [6.1;15.0] ^#^	7.2 [3.7;11.7]	1.06 (1.01;1.12)	0.016
Absent or decreased peristalsis (n [%])	16 [27.6] ^#^	23 [17.8] ^+^	1.76 (0.85;3.65)	n.s.
*Twenty-four-hour oesophageal pH-monitoring*				
DeMeester score (median [IQR])	24.6 [12.1;45.2]	29.7 [21.0;44.6]	0.99 (0.79;1.01)	n.s.
Total relax. time (%, median [IQR])	6.7 [4.0;11.5]	6.9 [4.6;12.5]	0.99 (0.67;1.47)	n.s.
Upright relax. time (%, median [IQR])	7.2 [4.1;13.2]	8.3 [4.7;14.0]	0.99 (0.96;1.03)	n.s.
Lying relax. time (%, median [IQR])	3.1 [0.0;12.8]	3.4 [0.6;8.6]	1.01 (0.97;1.04)	n.s.
*Barium esophagogram*				
Hiatal hernia (n [%])	32 [49.2]	92 [70.8]	0.52 (0.28;0.96)	0.035
Reflux (n [%])	28 [43.1]	68 [52.3]	0.81 (0.44;1.42)	n.s.

*n.s.* not significant; ^#^ n = 58 (seven patients did not tolerate manometry), ^+^ n = 129 (one patient did not tolerate manometry), p p-value; all parameters were tested using univariate logistic regression models.

HbA1c levels in patients with T2D did not correlate with LES pressure, relaxation time, or peristalsis (r <0.30, p > 0.05 for all three Spearman rank correlations).

### Diabetes treatment-specific analyses

Symptoms, endoscopic, pH, and manometric parameters in patients with T2D were also analyzed according to the diabetes-specific therapy the patients were receiving. There were no differences in all parameters between those patients with dietary restrictions only (n = 10), those with dietary restrictions and oral hypoglycaemic agents (n = 43), and those who were also insulin-dependent (n = 12), apart from epigastric pain. This symptom was reported less frequently in patients on oral medication (51.2%) than in patients with dietary restrictions only (80%) and those with additional insulin therapy (83.3%, p = 0.045).

## Discussion

Diabetes is a major health problem, as it is an important contributor to various other diseases and its incidence still continues to rise. Despite the fact that its role as a risk factor for GERD has been demonstrated and that GERD itself is of high clinical relevance, little light has been shed on the connection between the two diseases [3,4,6]. For the first time, the present study characterizes GERD patients concurrently affected by T2D using well-established diagnostic methods for oesophageal testing.

The individual symptom load differs between diabetics and non-diabetics [19]. In our study, dysphagia and globus sensation were found significantly more frequently in GERD patients with T2D. In the course of GERD, dysphagia may be caused by decreased oesophageal motility or by peptic oesophageal stricture due to acid reflux [20,21]. In our study population, impaired peristalsis, as assessed by manometry, was somewhat more frequent among patients with T2D. Moreover, dysphagia has been shown to be related to the presence of high LES pressure in patients with a hypertensive LES [22,23], which is also true for our investigation.

Globus sensation has been reported to be a common functional symptom due to inflammation of the throat caused by GERD [24,25]. In our study, however, there was no significant difference in DeMeester scores between the two groups. Diabetics used antidepressants more frequently than non-diabetics. Psychological impairment has been discussed as another possible factor contributing to globus sensation [26]. The approximately two-fold higher prevalence of laryngeal lesions in patients with T2D may also contribute to the increased prevalence of globus sensation. The exact etiology of globus sensation, however, remains incompletely understood [27,28].

Epigastric pain and heartburn are known to be the most frequent GERD-associated symptoms, occurring in up to 90% of patients with proven T2D [29]. Similar results have been found in the present investigation. The higher prevalence of heartburn, defined as “hypersensitivity” to acid reflux in the non-diabetes group, may be explained, at least partly, by diabetic neuropathy [4,8,30]. Moreover, the higher rates of hiatal hernia in the non-diabetes group in our study may have also contributed to the higher heartburn rate [31]. However, epigastric pain and heartburn are not necessarily associated with GERD. Studies that deal with symptoms only, without oesophageal testing, cannot verify the actual presence of GERD [3-5,9,10]. Thus, a direct comparison between the results of these studies and our findings is not valid.

Regurgitation, a major problem for many reflux patients, is predominant in non-diabetics and within the range described for this population [8]. Increased rates of hiatal hernia in the non-diabetes group may have had an impact on this finding [31]. For patients with T2D, however, the regurgitation rate of about 48% was much higher in our study compared to 16% in a previous series of type 2 diabetics [32]. Impaired peristalsis was found more frequently in patients with T2D, which may be another explaination for our findings.

Microaspiration of the gastro-oesophageal refluate and subsequent airway inflammation, are the major mechanisms that cause respiratory symptoms in GERD patients [33-35]. Although the overall prevalence of respiratory symptoms did not differ between T2D patients and non-diabetics, it is noteworthy that laryngeal lesions, including leukoplakia and ulcerations, were observed more frequently in patients with T2D. Recently, epidemiologic evidence has suggested that cancer incidence is associated with DM, as well as certain diabetic risk factors, including obesity [36-38]. Our goal, however, was not the long-term observation of possible precancerous lesions. Nevertheless, to the best of our knowledge, this is the first report describing extra-oesophageal reflux symptoms in diabetic GERD patients.

Overall, the summed scores of GERD symptoms were comparable in diabetics and non-diabetics in our study. Similar results have been published earlier [19,39]. Likewise, GERD-specific quality of life was comparable in diabetic and non-diabetic patients although there were some minor differences in health related quality of life [40]. All in all, data on the prevalence of reflux symptoms vary widely within T2D patients, ranging from 25.3% to 45.0% [3,39]. In contrast to other studies, we did not screen for GERD-related symptoms in diabetics since these symptoms were an inclusion criterion in our study.

Notably, the duration of T2D and the presence of neuropathia had no major impact on GERD symptoms in our investigation [41,42]. In the present series, this was not the case. This is probably due to the fact that (i) patients with T2D were under adequate glycaemic control, has already been pointed out earlier [3,9,43]. Moreover, (ii) the duration of GERD symptoms did not differ compared to non-diabetics.

Hiatal hernia rates were relatively high in both groups. Patients referred to our motility laboratories for GERD diagnostics are usually those who still suffer from GERD-related symptoms despite PPI intake. In general, hiatal hernia is the major reason for persisting complaints [44]. This could possibly be some kind of ‘pre-selection’ of our patients. However hiatal hernia rates were more frequent in non-diabetics. This is a surprising result considering that higher BMI levels are known to be a risk factor for the presence of hiatal hernia, and that higher BMI levels were found in patients with T2D in our study [37,45]. Nevertheless, our results are in accordance with a previous report demonstrating that, in morbidly obese patients, the rate of hiatal hernia was inversely correlated with glycaemic control [46]. In diabetics, delayed gastric emptying and decreased proximal retention of meals have been reported [47]. Furthermore, an inverse correlation between proximal gastric volume and the formation of a sliding hiatal hernia has been described [48]. Thus, altered intragastric meal distribution in patients with T2D may contribute to a lower prevalence of hiatal hernias in diabetic patients.

Higher BMI levels, as found in the T2D patients in our study, are an established risk factor for the development of GERD, and, thus, for esophagitis [49]. Notably, histological rates of esophagitis and Barrett’s oesophagus, as well as low-grade and high-grade dysplasia, were somewhat higher in T2D patients compared to controls. Although these results failed to reach statistical significance, which is probably due to the relatively low number of patients, one has to emphasize the possible clinical importance of such results, because, for example, low-grade dysplasia was more than twice as high in diabetics than in non-diabetics (10.8% vs. 3.8%). It has already been suggested that visceral fat, another contributing factor to T2D, was associated with Barrett’s esophagitis, and with increased esophageal inflammation and high-grade dysplasia in subjects with Barrett’s esophagitis [50]. Moreover, since patients with T2D may have a lower oesophageal sensitivity, as suggested by the decreased heartburn rates, GERD and, in consequence, precancerous lesions, might be diagnosed later than in non-diabetics. However, the primary goal of our study was not to prove the increased risk of T2D patients for developing oesophageal adenocarcinoma, but to describe T2D-related differences in the symptom load and the pathophysiology of GERD. Consequently, surveillance studies will be necessary in order to explore the cancerous potential of T2D in GERD patients.

Our results showed significantly higher rates of helicobacter pylori infection in patients with T2D. This is in accordance with the published literature, as diabetics, especially those with poor glycaemic control, are more prone to infections [8,43]. Nevertheless, other studies reported even higher percentages of helicobacter pylori infection, with differences between diabetics and non-diabetics of up to 80% versus 30%, respectively [51,52]. The discrepancy between these results and our findings may be explained by the fact that many of our patients had been pretreated by eradication therapy.

Hyperglycaemia decreases LES pressure in diabetics, with up to 92% of patients having resting LES pressure between 0 and 8mmHG [30]. Consequently, lower LES pressure predisposes to GERD [8]. In our investigation, however, T2D patients had significantly higher LES pressure levels compared to non-diabetics. Various studies have dealt with LES pressure in diabetic subjects [30,53]. However, these data are only partly comparable to our results, since all of these studies included diabetic patients without a history of GERD.

DeMeester scores were lower in T2D patients, although not statistically significant. The higher prevalence of hiatal hernia in the non-diabetic group and the significantly lower LES pressure may account for this finding. In addition, high helicobacter pylori infection rates could have ameliorated gastric secretion [8].

Notably, our data show no significant correlations between HbA1c levels and LES pressure, relaxation time, or peristalsis. Gastrointestinal symptoms and complications do not always correlate with the duration of DM and glycaemic control [43]. To date, no single risk factor for GERD in T2D has been identified, as the aetiology of GERD is multifactorial [43].

The limitation of our study is the retrosprective design, although all data were derived from a prospective database. The relatively low number of patients might be considered another drawback of the study. To the best of our knowledge, however, this is the largest investigation on proven GERD in T2D.

## Conclusion

T2D GERD patients (i) exhibit other types of symptoms than do non-diabetics, especially dysphagia, globus sensation, and, at least to some extent, extra-oesophageal symptoms with higher rates of proven laryngeal lesions; (ii) are infected with helicobacter pylori more frequently; and (iii) show higher LES pressure levels; and (iv) lower rates of hiatal hernia. The results may have some implications for diagnostic and therapeutic strategies in diabetic GERD patients. Since the incidence of the most frequently encountered GERD symptom, namely heartburn, is lower in patients with T2D, other signs and symptoms should lead to the suspected diagnosis of GERD. Particularly with regard to possibly higher rates of dysplasia and Barrett metaplasia in T2D GERD patients, the diagnostic work-up in diabetic patients could be of the utmost importance.

## Abbreviations

GERD: Gastro-oesophageal reflux disease; T2D: Diabetes mellitus type 2; LES: Lower oesophageal sphincter; BMI: Body mass index; DM: Diabetes mellitus.

## Competing interest

All authors agree to the manuscript’s submission, confirm that our manuscript is not submitted or being considered for publication elsewhere in any until your editorial decision, and agree that if accepted the paper will not subsequently be published in the same or similar form in any language without the written consent of the publisher. All authors have read and approved the final version of the manuscript.

All authors declare that they have no commercial interest, financial interest, and/or another relationship with manufacturers of pharmaceuticals, laboratory supplies, and/or medical devices or with commercial providers of medically related services. There was no funding.

## Authors’ contributions

RP contributed to the project’s conception, participated in acquisition of data and drafted the manuscript. OR and GS performed the statistical analysis. MG and JO performed the statistical analysis and drafted the manuscript. JL and FMR contributed to the project’s conception. WHE participated in acquisition of data. MG contributed to the project’s conception. CN contributed to the project’s conception, data and drafted the manuscript. All authors read and approved the final manuscript.

## Pre-publication history

The pre-publication history for this paper can be accessed here:

http://www.biomedcentral.com/1471-230X/13/132/prepub

## Dedication

This study is dedicated to Prof Dr Michael Gadenstätter (1963–2013), who recently passed away unexpectedly. We will keep him in memory as a friendly end engaged chief of the Department of Surgery, General Hospital of Krems, Austria. Professor Gadenstätter substantially contributed to GERD literature and antireflux surgery. We keep him in kind remembrance.
